# Do metabolic syndrome and its components have an impact on bone mineral density in adolescents?

**DOI:** 10.1186/s12986-016-0156-0

**Published:** 2017-01-04

**Authors:** Valéria Nóbrega da Silva, Luciana Nunes Mosca Fiorelli, Carla Cristiane da Silva, Cilmery Suemi Kurokawa, Tamara Beres Lederer Goldberg

**Affiliations:** 1 Department of Pediatrics, Discipline of Adolescent Medicine, Postgraduate Program in Gynecology, Obstetrics, and Mastology, Botucatu School of Medicine, UNESP, São Paulo State University, Botucatu, São Paulo Brazil; 2 Department of Physical Education, University of North Paraná, Jacarezinho, Paraná Brazil; 3 Clinical and Experimental Pediatric Research Center, Department of Pediatrics and Postgraduate Program in Tropical Disease, Botucatu School of Medicine, UNESP, São Paulo State University, Botucatu, São Paulo Brazil

**Keywords:** Metabolic syndrome X, Abdominal obesity, Waist circumference, Bone mineral density, Hypertriglyceridemia, Adolescents

## Abstract

In recent years, there has been growing concern about the occurrence of metabolic syndrome (MetS) at an early age and its effects on bone mass in adolescents. Adolescence is considered a critical period for bone mass gain. Impaired bone acquisition during this phase can lead to “suboptimal” peak bone mass and increase the risk of osteopenia/osteoporosis and fractures in old age. The objective of this review was to perform a critical analysis of articles that specifically focus on this age group, evaluating the influence of MetS and its components on bone mineral density in adolescents. A possible relationship between this syndrome and bone mass has been demonstrated, but the number of studies addressing this topic in adolescents is small. Despite the scarcity of evidence, the results of those studies show that Metabolic Syndrome is negatively correlated with bone mass and also that some components of MetS are negatively correlated with bone mineral density in adolescents. However, the associations between MetS and bone mass development need to be further explored in the age group corresponding to adolescence. Further good-quality studies are necessary to complement the understanding of this relationship.

## Background

Metabolic syndrome (MetS) is a clinical condition characterized by a combination of abdominal obesity, altered glucose metabolism, dyslipidemia, and arterial hypertension. This combination of metabolic alterations predisposes affected individuals to the development of cardiovascular diseases and type 2 diabetes mellitus [1, 2]. In addition to the cardiovascular problems extensively documented in the scientific literature, previous studies have shown a possible relationship between MetS and bone mass, however the results are still inconsistent [3–7].

In adults, studies have shown a negative correlation between MetS and bone mineral density (BMD) [3, 6, 8, 9]. These findings have been questioned in a recent systematic review with meta-analysis, suggesting that BMD at different sites does not differ between adult men and women with and without MetS [10]. However, the authors suggested caution in the interpretation of the results and indicated the need for prospective studies [10]. Among Caucasian Dutch adults, an association with femoral BMD was observed in older women with MetS, even after adjustment for confounding parameters such as body mass index (BMI), age and lifestyle, while no such association was found in older men. Analysis of the cohort showed a lower odds ratio of osteoporosis and fractures in both genders [11]. Unfortunately, that study only evaluated the femoral region [11]. However, the authors emphasized that the presence of MetS could increase fractures of the humerus and ankle. Another hypothesis to explain the diversity of the results of already published studies is the fact that the presence of MetS associated with a reduction in BMD was demonstrated for other ethnic groups and younger individuals, and therefore depends on the population studied. With respect to adolescence, literature reviews on the relationship between MetS and BMD are sparse [4, 5, 7].

Adolescence is considered a critical period for bone mass gain. The greatest gains in bone occur during this phase when peak bone mass is reached [12]. Impaired bone growth during this period can lead to “suboptimal” peak bone mass and increase the risk of developing osteopenia/osteoporosis and fractures in old age [13, 14].

In view of these considerations, the objective of the present study was to review the possible effects of MetS and its components on BMD in adolescents.

### Search strategy and article selection

A literature review was performed. The following electronic databases were searched for articles published over a period of 10 years (January 2004 to June 2015), without language restrictions: Scientific Electronic Library Online (SciELO), Latin American and Caribbean Health (LILACS), Medline, PubMed, Scopus, and Cochrane Library.

The following MeSH terms were used alone or in combination: “metabolic syndrome or metabolic syndrome X or plurimetabolic syndrome or MetS or cardiometabolic risk factors or cardiovascular disease or cardiovascular disease risk factors” or “abdominal obesity or waist circumference” or “hypertriglyceridemia” or “HDL cholesterol” or “diastolic blood pressure or systolic blood pressure” or “fasting glucose” and “bone densities or bone mineral density or bone mineral densities or density, bone mineral or bone mineral content or bone mineral contents or bone density or physiological calcification or bone mineralization”, and “adolescents or adolescence or teens or teen or teenagers or teenager or youth or youths”.

Human studies on the association between MetS and bone mass, regardless of sample size, were included if they met the following criteria: overweight adolescents with cardiometabolic risk factors (waist circumference, HDL cholesterol, triglycerides, diastolic blood pressure, systolic blood pressure, fasting glucose) and aged ≥ 10 years. We considered cohort studies as well as cross-sectional studies. The latter is an anticipation of not finding many prospective studies with a control group. BMD or bone mineral content (BMC) or bone area was measured by dual-energy X-ray absorptiometry (DXA). Comments, letters, and articles containing only the abstracts without the full text were excluded.

At the time of the initial database search twenty studies were identified and selected to be included in the review based on the predefined inclusion criteria. After reading the titles and abstracts, 14 studies that did not meet the inclusion criteria for the review were excluded: age of the study population < 10 years, absence of groups of adolescents with excess weight, lack of comparison between eutrophic and overweight adolescents, absence of cardiometabolic risk factors, use of other methods for the evaluation of BMD, BMC or bone area, and presence of other diseases or syndromes associated with MetS.

### Metabolic syndrome and bone mass

Literature reviews on the relationship between a diagnosis of MetS and risk factors during puberty and BMD in adolescents are sparse [4, 5, 7, 14–16]. Table 1 shows the results of some studies on the topic found in the scientific literature according to date of publication and interest in investigating the risk factors of metabolic syndrome, with bone mass evaluated through densitometry in adolescents.

**Table 1:** Studies evaluating the association of metabolic syndrome or a separately altered component with BMC, BMD in adolescents

Study	Country	Sample	Design	Outcome measures	Results - association between MetS or risk factors and bone mass
Afghani et al. [15]	California/USA	184 overweight (8 to 13 years)	Cross-sectional	BMC using DXA	Insulin resistance↓ body BMC
Pollock et al*.* [16]	Georgia/USA	140 overweight (7 to 11 years)	Cross-sectional	BMC and BMD using DXA	Pre-diabetes ↓BMC and BMD Hyperinsulinemia ↓BMC and BMD
Pollock et al*.* [4]	Georgia/USA	143 overweight adolescents with cardiometabolic risk factors (14 to 18 years)	Cross-sectional	BMC using DXA	↑HDL-c ↑BMC ≥2 MetS components ↓BMC Increased WC ↑visceral adipose tissue, fasting insulin and HOMA-IR ↓total body BMC
Lawlor et al. [5]	United Kingdom	2035 adolescents (15 years)	Cross-sectional	BMD and BMC using DXA	Hypertriglyceridemia ↑BMD, BMC and in boys Reduced HDL and insulin resistance ↓total body BMD, BMC
Lee et al. [14]	South Korea	618 adolescents (10 to 19 years)	Cross-sectional	BMC using DXA	HOMA-IR ↓BMC in boys
Nóbrega da Silva et al. [7]	Brazil	270 overweight adolescents (10 to 16 years)	Cross-sectional	BMD using DXA	MetS (+)↓BMD/kg weight lumbar spine, left proximal femur, total and subtotal body Increased WC ↓BMD (lumbar spine and total body) in both genders Hypertriglyceridemia ↓BMD (lumbar spine and total body) in girls.

*BMC* bone mineral content, *BMD* bone mineral density, *MetS(+)* with metabolic syndrome, *WC* waist circumference

Afghani et al*.* [15] studied a cohort of overweight Latin American children and adolescents with a family history of type 2 *diabetes mellitus*. The authors observed that total body BMC was negatively correlated with the presence of markers of insulin resistance; however, they did not evaluate the effects of other MetS components on bone mass. Similar findings have been reported by Pollock et al*.* [16] who evaluated total body BMC and BMD in overweight prepubertal children with and without pre-diabetes. These authors observed a significant reduction in both BMC and BMD in the group with pre-diabetes and hyperinsulinemia. In a subsequent study, Pollock et al. [4] compared bone mass between overweight adolescents (14 to 18 years) with and without cardiometabolic risk factors. In that study, BMC was reduced by 5.4% in adolescents that had at least one component of MetS compared to those without any risk factor, and by 6.3% when two or more risk factors were present. The authors found no correlation between elevated triglyceride levels and BMC; however, increased waist circumference, increased visceral fat tissue, fasting insulin and homeostasis model assessment of insulin resistance (HOMA-IR) showed significant negative correlations with total body BMC. In contrast, HDL-cholesterol and total energy intake were positively associated with BMC. In a comment on the study of Pollock et al. [4], Kalkwarf [17] emphasized that these findings were only detected after adjusting total body BMC for fat-free soft tissue mass, assuming that, if the crude BMC values were not adjusted, they would misleadingly indicate that bone mass increases with increasing body weight, a situation previously reported in the literature.

The results published by Mosca et al. [18] corroborate these findings by demonstrating a negative correlation between BMD of overweight, obese or extremely obese adolescents and the percentage of fat mass determined by DXA. The authors concluded that the higher the body fat percentage of adolescents, the lower the BMD and BMC [18]. Complementing these considerations, it should be emphasized that the prevalence of MetS is higher among extremely obese adolescents [19].

Lawlor et al. [5] conducted a cross-sectional study involving a sample of 2305 adolescents in an attempt to demonstrate associations of markers related to insulin resistance (fasting glucose and insulin), triglycerides and HDL-cholesterol with BMD. Controlling for the covariates age, height and pubertal stage in multivariate analysis, the authors found no significant correlation between fasting glucose and BMD. Triglycerides were positively correlated with BMD, BMC and bone area in boys. HDL-cholesterol showed an inverse correlation with BMD, BMC and bone area in both genders. After adjusting for fat mass, fasting insulin in boys was inversely correlated with bone mass. According to these authors, this finding after adjustment for fat mass should be treated with caution and further prospective studies are needed to replicate and explore the results.

A recent study published by Brazilian researchers found a reduction in BMD at different sites in overweight adolescents with MetS when compared to adolescents in the same nutritional condition, but without MetS. Furthermore, adolescents with two or more risk factors for MetS exhibited a significant reduction in bone mass compared to those with no or only one risk factor. Among the MetS components, waist circumference was the determinant factor for BMD reduction [7].

It can be observed that the majority of the studies found were transversal with evaluation of bone densitometry through DXA. In relation to bone histomorphometry, quantitative histological evaluation of calcified bone biopsy performed to obtain information on remodeling and bone structure, a recent experimental study was detected, being constructed to evaluate, among other parameters, the bone histomorphometry of the tibia and vertebra of experimental animals, in the growth phase and mature, who received a diet rich in saturated fat and sucrose (HFS), against animals considered controls, who received diet chow. The authors concluded that the animals submitted to HFS developed a phenotype characterized by excess visceral fat, which presented through increased abdominal circumference and body weight, non-alcoholic hepatic steatosis, and a 334% increase in basal insulinemia in those in the growth phase and 86% in mature rats, indicating insulin resistance, alterations that resembled metabolic syndrome comorbidities. The mature rats maintained in this scheme also developed pressure alterations and a reduction in HDL cholesterol. In addition, after 27 weeks of follow-up, the animals presented a reduction in active osteocalcin (OC). These animals also presented reduced calcemia and increased phosphataemia, in addition to a reduction in the bone surface, thickness of the osteoid, with more important alterations in the vertebral bones than in the tibia, since these suffered the impact of the load. The authors proposed that metabolic syndrome caused by dietary HFS intake increased the porosity of the cortex of the tibia [20].

### Puberty and evaluation of bone mass

The period of puberty is characterized by the occurrence of a fundamental process, i.e., the maximum acquisition of BMC [12, 21–24]. Bone tissue is composed of cells, called osteoblasts and osteoclasts, minerals (calcium and phosphorus), and an organic matrix consisting of collagen and non-collagen proteins. Osteoblasts synthesize and mineralize the protein matrix, while osteoclasts promote bone resorption, maintaining the bone tissue in a constant process of remodeling. During childhood and adolescence, the rate of bone formation exceeds that of bone resorption, favoring bone acquisition [22, 25]. Approximately 40 to 45% of adult bone mass is acquired during adolescence [26]. In this respect, periods of skeletal growth, especially during adolescence, are fundamental for peak bone mass acquisition and to reduce the risk of developing skeletal morbidities such as osteopenia/osteoporosis and fragility fractures in old age [13, 14, 21, 27]. In both genders, peak bone mass acquisition occurs around seven or eight months after maximum longitudinal bone growth (growth spurt) as a result of high concentrations of hormones [22, 24, 25, 28, 29].

The occurrence of low bone mass in children and adolescents has been identified in recent years and has raised the interest of the scientific community [4, 30]. On the one hand, there is growing awareness that bone mineral mass acquired at the end of growth and its development are crucial for reducing the risk of osteoporosis in the future. On the other hand, osteoporosis is increasingly more prevalent and also occurs in young patients, since BMD in these age groups depends on peak bone mass acquired by the end of the second decade of life [31].

The increase in recent decades in the frequency of fractures in childhood from 35% to 65% has raised concerns that the current lifestyle is compromising early bone health [30]. Bone densitometry detects bone mass losses of less than 5%. The interpretation of DXA results in the pediatric population is a challenge because of the changes in bone size and geometry that occur during growth and child development. Adequate interpretation of the results should take into consideration skeletal maturity, pubertal development, ethnic background, weight, and height of the patient [25].

### Metabolic syndrome, risk factors and bone mass

The presence of central obesity, defined by increased waist circumference, and at least two of the following four criteria are necessary for the diagnosis of MetS: triglyceride elevation (≥150 mg/dL); reduction in HDL-cholesterol (<40 mg/dL); arterial hypertension (SBP ≥ 130/DBP ≥ 85 mmHg); and fasting hyperglycemia (blood glucose ≥100 mg/dL), or previously diagnosed type 2 *diabetes*. [1].

According to Nóbrega et al. [7], the prevalence of MetS was 14% among 271 adolescents, being 13.29% and 15.93% for female and male adolescents respectively. In relation to the components of MetS, there was a higher prevalence in the increase in waist circumference (67.82%), reduction in HDLc (32.09%), Hypertension (22.62%), Hypertriglyceridemia (19.78%), and Hyperglycemia (3.37%).

Within the global context of an increasing prevalence of obesity and other cardiovascular risk factors in children and adolescents, the role of MetS is particularly important, however, despite this, it has been little investigated in the literature. In addition to the health problems already documented in the scientific literature, previous studies have provided indicators of a relationship between MetS and bone mass in the young and adult population, but the results are still inconsistent [3, 4, 6, 7].

#### Waist circumference and bone mass

**Fig. 1:**
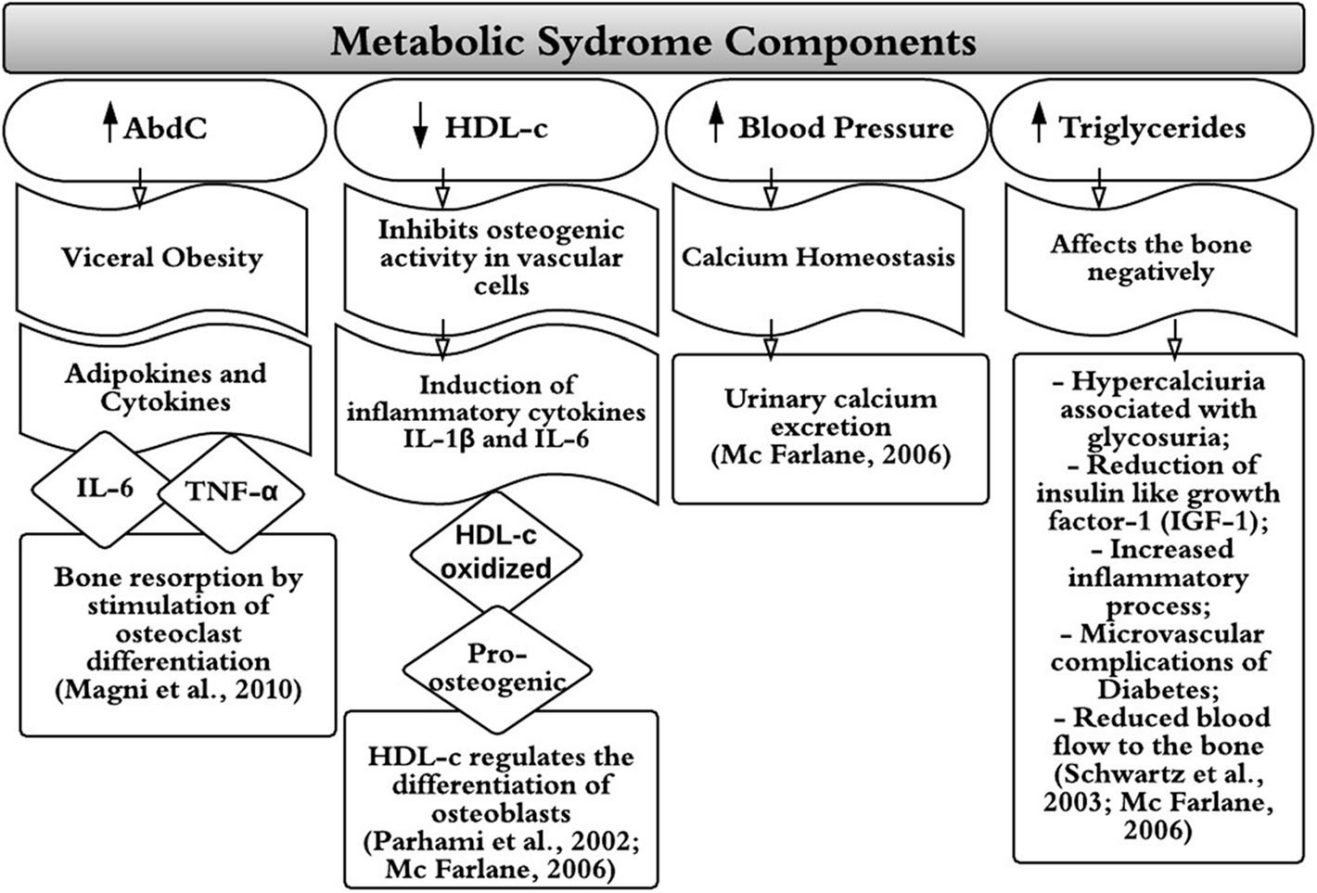
Metabolic Syndrome and decrease in bone mass with explanations

Several mechanisms of action can be proposed for the repercussions of MetS components on bone mass (Fig. 1).

Visceral obesity determined by the evaluation of waist circumference is an important factor that contributes to the negative association with BMD, suggesting that fat, particularly visceral fat, is deleterious to bone mass [4, 18, 32, 33]. In addition to its role in the storage and mobilization of lipids, visceral fat has also been considered an endocrine organ that releases adipokines and cytokines, including proinflammatory molecules such as interleukin 6 (IL-6) and tumor necrosis factor alpha (TNF-α) [34]. There is consensus that IL-6 and TNF-α promote bone resorption by stimulating the differentiation of osteoclasts [35] (Fig. 1). Furthermore, the accumulation of visceral fat, lipid profile alterations and blood pressure changes, the main components involved in MetS, are significantly correlated with low serum levels of osteocalcin in the adult population, which is considered a sensitive marker of bone formation [36, 37].

In obese children, circulating osteocalcin levels were found to be lower than in eutrophic children and were positively correlated with the presence of insulin resistance and negatively with serum leptin concentration [38]. Recently, Kim et al. [39] investigated the association of serum total osteocalcin with obesity and MetS in Korean children and identified significantly lower osteocalcin levels in overweight and obese children compared to the mean values obtained for eutrophic children. The authors observed a negative correlation between osteocalcin and adiposity. This significant negative correlation between osteocalcin and body fat percentage was maintained even when the variables were adjusted for gender and age. The authors also found significant differences in osteocalcin and MetS between groups. Participants without MetS components had significantly higher osteocalcin levels than those with one or two risk factors for MetS, in addition to increased waist circumference.

Regarding other factors with a negative impact on bone metabolism, elevated leptin concentrations are detected in obese children and adolescents based on the fact that they accelerate bone resorption and reduce bone formation. The consequences are alterations in the bone microstructure of these individuals and an increased fracture risk [40, 41]. Two mechanisms of action of leptin on bone mass have been proposed: one through sympathetic activity in which leptin binds to hypothalamic receptors, increasing the expression of RANKL and consequently bone resorption, and the other through cocaine- and amphetamine-regulated transcription, which results in the inhibition of RANKL [42, 43].

#### Dyslipidemia and bone mass

Knowledge of the mechanisms related to the participation of HDL-cholesterol in the calcification of vascular lesions can be applied to the understanding of its effect on skeletal formation [44]. A possible explanation of the effect of HDL-cholesterol is that this lipoprotein inhibits the osteogenic activity of vascular cells by inducing inflammatory cytokines such as IL-1β and IL-6. Oxidation of HDL-cholesterol renders it pro-osteogenic, suggesting that HDL-cholesterol regulates the differentiation of osteoblasts, the cells involved in the formation of bone tissue [44] (Fig. 1). Also regarding the detection of hypercholesterolemia, Abramowicz et al. [45] observed negative correlations of lumbar spine BMD and total body BMC with increased total cholesterol in overweight/obese girls, but not in boys. The authors suggested that the risk of cardiovascular events and osteoporosis later in life would be higher in these girls.

In adults, a previous study highlighted that elevated levels of triglycerides are negatively correlated with femoral neck BMD in postmenopausal women and are detrimental to bone mass [3]. Thus, it appears that high levels of triglycerides are also harmful to bone in adolescents [7]. A negative correlation with BMD at all sites analyzed has been observed in adolescent girls aged 10 to 16 years [7].

Bredella et al. [46] focused on bone marrow fat since this is the site of stem cell differentiation into osteoblasts, the cells responsible for bone formation. In that study, the authors used magnetic resonance spectroscopy to accurately measure bone marrow fat content in 106 obese subjects ranging in age from 19 to 45 years. The study revealed high levels of bone marrow fat in subjects with increased liver and muscle fat content and these results were independent of body mass index, age and exercise status. Furthermore, HDL-cholesterol was associated with a lower risk of heart disease and was inversely associated with bone marrow fat content. The authors observed that triglycerides were positively correlated with bone marrow fat, possibly because this type of fat stimulates osteoclasts, the cells that degrade bone tissue. The authors highlighted the fact that high levels of bone marrow fat increase the risk of fracture [46].

#### Arterial hypertension and bone mass

Arterial hypertension can be associated with abnormal calcium homeostasis, resulting in an increase in the urinary excretion of this mineral [44] (Fig. 1). An association between low BMD and arterial hypertension has been described in men and in postmenopausal women [47, 48]. However, Pollock et al. [4] found no association between arterial hypertension and bone mass in overweight adolescents. Pludowski et al. [49] reported the presence of lower bone mass evaluated based on total body BMC in 94 adolescents (21 girls and 73 boys) with primary hypertension compared to a control group without hypertension (*n* = 562) after adjusting for body weight and lean mass. Although reduced, BMC was within the physiological range. The hypothesis of that study was based on the fact that, in adults, both events (hypertension and osteoporosis) result from a reduction in physical activity and poor eating habits such as diets rich in sodium and low in calcium. Afgahani and Goran [50] suggested hypertension to be a risk factor for osteopenia observed in 187 overweight Latino children and adolescents. This risk was more evident in adolescent boys in late puberty.

#### Hyperglycemia and bone mass

There is no consensus in the literature regarding the association between altered glucose levels and BMD during adolescence. The mechanisms have not yet been fully unraveled and studies suggest that abnormal glucose levels may negatively affect bone through multiple pathways, including an increase in the concentrations of advanced glycated end-products, hypercalcuria associated with glycosuria, a reduction in insulin-like growth factor 1, an increase in inflammatory processes and cytokines, and microvascular complications resulting from diabetes and the consequent reduction in blood flow to bone [44, 51] (Fig. 1). Within this context, further studies are needed to explore these associations [44].

## Conclusion

The associations between MetS and bone mass development in adolescents have not been widely explored and the results have been poorly enlightening. Prospective studies are therefore necessary to explore these associations since the presence of low BMD during adolescence can significantly compromise peak bone mass acquisition, with a consequent risk of fractures during this phase of life and bone fragility later in life.

## Abbreviations

BMC: Bone mineral content
BMD: Bone mineral density
BMI: Body mass index
DBP: Diastolic blood pressure
DXA: Dual energy X-ray bone densitometry
HDLc: HDL cholesterol
HOMA-IR: Homeostasis model assessment of insulin resistance
IDF: International Diabetes Federation
MetS: Metabolic syndrome
SBP: Systolic blood pressure
WC: Waist circumference
